# Cleft of the Hard and Soft Palates*Read before Philadelphia Academy of Surgery.

**Published:** 1894-02

**Authors:** J. Ewing Mears

**Affiliations:** Philadelphia, Pa.


### Southern Medical Record.

A MONTHLY JOURNAL OF MEDICINE AND SURGERY.

VOL. XXIV. ATLANTA, GA., FEBRUARY, 1894. No. 2.

### Original Articles.

### CLEFT OF THE HARD AND SOFT PALATES.*

BY J. EWING MEARS, M. D., PHILADELPHIA, PA.

An experience extending over a period of twenty-five years, in which time I have treated by operative procedures upward of one hundred cases of deformities of the palate, has afforded me an opportunity to study the conditions involved, the measures for relief, and the value of the results obtained. The cases under treatment have included all forms, I believe, of congenital and acquired cleft—deformity limited to the soft palate, partial and complete in its extent; of the hard palate—partial and complete, including involvement of intermaxillary bone, and the upper lip, with either single or double cleft. With regard to the cleft of the soft palate, I have observed that in nearly all cases of complete cleft there has coexisted a cleft in the soft palate, implicating to a slight extent the horizontal plates of the palate bone. I soon learned that this condition had an important relation to the success obtained in effecting closure, and I shall refer to it when discussing operative measures.

Of acquired cleft I have met with various forms—from simple perforation, by a small opening, to almost entire obliteration of the

*Read before Philadelphia Academy of Surgery.

soft and hard palates, with destruction of portion of the osseous structures of the nasal cavities. In one instance there was not only a fissure in the median line, but the lateral portions were united by firm adhesion to the posterior wall of the pharynx.

In congenital cleft of the hard palate I have met with instances in which the fissure being bilateral, there has been more or less deficiency of the vomer, and in other cases the fissure being limited largely to one side, the vomer, somewhat deflected, has been found in articulation with the side less involved, thus permitting communication between one nasal fossa and the oral cavity only.

In the study of the cases under treatment I have been interested in arriving at satisfactory conclusions as to the cause concerned in the production of the deformity, and to what extent hereditation and maternal impressions take part. During the summer of 1876 a lady from the far West came to my office for consultation. As soon as she addressed me I thought I recognized by the nasal articulation and guttural tone of voice a cleft of the palate, and remarked: "I know why you have come to me for consultation; you have a cleft palate." She replied: "I have not, but I have the articulation of one having a cleft palate, and I have come to you for relief." On examination, I found there existed a marked shortening and rigidity of the soft palate which prevented its contact with the posterior wall of the pharynx and thus led to the production of the nasal tone of voice. By division of the tensor palati and the palato-glossi and pharyngei muscles I relieved the tense condition of the velum and secured its elevation so that it approached quite nearly in contact with the posterior wall of the pharynx and very materially improved the articulation. The patient had recently married. A few years after she brought to me her first-born child, which had a complete cleft of the soft, with partial cleft of the hard palate, upon whom I operated with good success.

A man, upward of fifty years, consulted me for cleft of the soft palate, the result of diphtheritic inflammation when a child. His mother dying during his childhood, his father married a second time, and he was a victim of a step-mother's jeers, as well as of her cruelty, on account of his unfortunate condition. Daily she would imitate his tone of voice. Her first child by the victim's father had cleft of the palate. Other instances I have met with in which the mother attributed the condition in the child to having conversed with a person who suffered from cleft palate, etc.

Whatever causal effect hereditation and maternal impressions may have, the observations made in Dublin Zoölogical Gardens some years since in the case of lion whelps have quite positively determined that the condition may be due to a want of proper nutriment in the mother during the period of gestation. Up to the time referred to it had been impossible for the authorities in charge of the garden to rear the young of the lion on account of the universal occurrence of cleft of the palate, which prevented the taking of nourishment to maintain life. The plan of giving to the mother during pregnancy ground bone and food containing the organic elements of bone was crowned with success, and within the past years a large revenue has been derived from the sale, to all parts of the world, of lions raised in this garden. The lion whelps in the Zoölogical Garden of this city have suffered in the same manner, and I have suggested to the superintendent to adopt the plan practiced in the Dublin Garden. During our conversation he stated an interesting fact, that the whelps of lions in traveling menageries did not suffer as those born of mothers confined in gardens, and he was disposed to account for the fact by the rough life led by them, as well as by the variety in the food, consisting of bones as well as of meat—sometimes more of the former than of the latter.

The conclusions which may be drawn from the consideration ot the above observation are to the effect that the fissures are due to a want of coalescence of the palatal processes and embryonic tissues during embryonic life, the absence of which union is not due to an infolding or reduplication of the membranes, but to a positive non-development caused by want of proper nutrition. Coalescence cannot take place because the parts are not in approximation.

The conditions presented by persons the subject of cleft palate are defective articulation, defective deglutition, and, as a rule, more or less chronic inflammation of the nasal and pharyngeal spaces. These conditions vary in degree in accord with the extent of the lesion, but I have observed as great an involvement of articulation where a slight notch only existed in the uvula as where both soft and hard palates have been fissured. The important function of the velum is, by its approximation to the posterior wall of the pharynx, to shut off the cavity of the mouth from the cavity of the nose, so as to prevent the passage of sounds and of food into the latter during articulation and deglutition. The muscles of the velum control by their action the opening and closing of the posterior nasal apertures, as those connected with the nasal cartilages regulate the opening and closing of the anterior nasal apertures. In cases of palate fissure in adults I have observed the use made by the anterior muscles in the effort to do the duty of the velum in so narrowing or contracting the anterior nasal openings as to prevent the escape of sounds or food. The chronic inflammation which exists in cases, as a rule, of extensive fissure is due to the exposure of the parts to the effect of the air which comes directly in contact with the surfaces without passing through the recesses of the normal nasal fossæ.

The object to be accomplished by treatment is to close the clefts. This can be done either by the adaptation of artificial appliances —obturators—or by operative procedures—staphylorrhaphy, when upon the soft palate; uranoplasty, when upon the hard palate. Since the introduction of these methods each has had its period of employment. Discouraged by the failures to obtain perfect results by operation, artificial appliances came into vogue and were employed to the exclusion of operative measures. These failing to satisfy the requirements, and especially failing to give comfort to the patient, lost favor and a revival of operative methods has occurred. In my experience I have never found the use of the obturator in cleft of the soft palate satisfactory. In cleft of the hard palate alone I have frequently advised its employment where the rudimentary palatal processes have been so narrow as to forbid operation, or where the opening was not very large. In some cases in adults of cleft of soft and hard palates I have closed the soft palate by operation, and have had adapted to the opening in the hard palate an obturator. In children I always advise closure by operation.

I have operated at various periods of life—from three years to fifty. I advise operation at an early age in order that the growth of the parts may be facilitated, and that the education of the child may receive proper attention. In children and young persons, especially in females, I use an anesthetic agent—a mixture of chloroform one part to ether two parts; the patient is placed or held in a sitting or semi-recumbent position. The mouth is held open by a gag which I devised and which has proven satisfactory, easy to introduce, readily held in place, and one which can be quickly removed. In paring the edges of the cleft I secure as large a raw surface as possible in order that the union may be strong. I have tried many forms of needles and varieties of sutures, and I have found the simplest the best. The instrument employed consists of a handle to which needles of various curves can be securely fastened. Silver wire sutures of medium size are used, a strong linen thread, double, being first deposited and the wire suture hooked into the loop and drawn through; the suture is deposited on one side at a a time. The needle is introduced some distance from the edge so as to include a sufficient amount of the tissue to hold the edges firmly in apposition without tension. When the sutures are all in position the edges are brought together and the muscles divided, which may be necessary to relieve all tension. The tensor palati is divided near to the hamular process, and the palato-glossi and palato-pharyngei as low down as they can be reached. In my later operations I have removed the sutures earlier, beginning on the fifth or sixth day to remove those affording the least support and completing removal by the seventh day. This plan has given better results. Liquid food, milk chiefly, is administered by a spoon whilst the sutures are in position. The patient gradually returns to the use of solid food.

In operations upon the hard palate the method of Sir William Ferguson has been latterly employed, having failed by other methods to secure good results. The steps of the operation consist in freshening the edges, depositing the sutures through openings made with the drill in the bony processes, division of the processes by the chisel or saw, drawing of the segments thus formed to the middle of the line, and securing them in place by twisting or shotting the wire. The section of the bone should be made so as to avoid wounding the vessels and nerves which come into the oral cavity through the posterior palatine canals, and which lie in a groove at the base of the alveolar process. Division of the soft palate downward is necessary to bring the segments in position without undue tension. The cavities formed by the transplantation of the segments are plugged with iodoform gauze, five per cent., which affords support and controls hemorrhage. On the third day the plug is removed, and as the spaces close they are gradually reduced in size.

*End-results. As to operative procedures on the soft palate.*—If careful examination has been made to detect slight notch in the hard plate and the bone has been divided, union in entire extent has occurred. In cases of long uvulæ non-union has sometimes occurred at the very tip. This has been remedied by subsequent operation on the end; if the length permits cut off.

*As to the hard palate.*—In certain cases slight necrosis of portions of the segments, caused by splintering at the time of section, has occurred and the pieces been removed. This has not interfered with the reparative process. In one instance the space on one side, left after drawing segments to the median line, was so large that it did not close completely—an obturator was successfully adapted.

*As to the restoration of function.*—In all cases deglutition has been markedly improved, rendered perfect except in a few instances of short and rigid vela in which hasty and careless efforts at swallowing are accompanied by the passage, especially of liquids, into the nasal cavities.

The improvement in articulation has varied greatly and the improvement has depended largely upon the size and conformation of the faucial and pharyngeal spaces. In a few it has been complete. I can recall at this time a lad who had complete cleft of palate, alveolar process, and upper lip. In infancy the cleft of the lip was closed by Professor S. D. Gross. At the age of twelve I closed the palate cleft, and in his case articulation was so perfect that no variation from the normal could be detected. A lady, twenty-eight years of age, had cleft complete of the soft with partial cleft of the hard palate. After operation she became a salaried member of a church choir, her voice being entirely free from nasal tone.

In many the improvement is partial but progressive, especially in children where pains are taken to educate them properly. In some cases where I have been able to retain charge of the patient I have rendered assistance, as growth took place, by subsequent division of the palate muscles, thus increasing the movement of velum.

In all cases, children and adults, the effect upon the *morale* has been good, and for this reason, if no other, the operation is justified.

DISCUSSION.

DR. JOHN B. ROBERTS: I have been much interested in Dr. Mears's paper. These are the cases in which we want the opinion of an expert, and a man who has seen as many of these cases as Dr. Mears is entitled to the respect that we give to an expert. I have had rather a large number of these cases recently. I see a good many cases in infants at the Woman's Hospital, and have been somewhat afraid to attack them. My feeling is that many of these cases are not very satisfactory, and in such young children I have been inclined to postpone operation, with the result that many of the cases have not returned. Thrice recently I have operated on hare-lips where there was also cleft palate, operation on which I postponed, feeling that there was not enough bony tissue. I was therefore anxious to hear how he managed these cases. The operations on the cleft lip have been somewhat unsatisfactory on account of the cleft alveolus failing to give support to the nose. In one case I cut through the alveolar process on both sides of the cleft and brought the parts over with a suture as suggested by Wyeth, but that case apparently is not going to do very well. I got the edges closer together, but they did not come in contact. Dr. Bower operated on a similar case, using a silk suture, and seems to have gained a great deal. The procedure does not close the palate, but it brings the anterior part together, but of course in these cases no attempt was made to close the posterior gap in either the soft or hard palate. I should be glad to hear what is Dr. Mears's experience in these cases. What does he do where there is cleft palate, with cleft of the lip and of the alveolus?

DR. W. W. KEEN: I am glad to find that Dr. Mears advocates the operative treatment which, as a rule, is by far the best. In some cases, however, where the cleft has been large, I have seen altogether the best results from the use of an obturator in the form of one made by Dr. Thorington, consisting of soft rubber with a soft rubber, and therefore flexible, palate.

There is one point on which I would ask Dr. Mears's experience and practice. In operating on cleft of the soft palate I have found no difficulty in securing union, except at one point, and that is where the soft palate joins the hard. The soft palate is very thick from a little above the tip up to near the point where it joins the hard palate. Here it becomes thinner, and in some cases almost translucent. In many cases where the rest of the palate has united, there has been left a little opening at this point, which I have had to close by a secondary operation, or by the use of nitrate of silver or the actual cautery. I should like to know if Dr. Mears has met with the same trouble, and how he has overcome it.

DR. R. H. HARTE: Has Dr. Mears tried the operation where one flap is transplanted from one side to the other in complete fissure of the hard and soft palate, and the raw surfaces then drawn together? I think it is known as Davies Colley's method.

DR. MEARS: In reply to Dr. Roberts I desire to say I have operated on cases where there has been a cleft of the palate, cleft of the alveolar process, displacement of the intermaxillary bone, the bone being turned up sometimes at right angles with cleft of the lip. My practice has been to break down the intermaxillary bone and bring it in place after freshening the edges, and then wiring the parts together. Where this bone has been taken away, as has sometimes been the case, I have divided the process on each side and drawn the detached portions over, approximating them in the center. I think that is the only method which can be practiced with any prospect of success. I close the hare-lip at an early age, sometimes at two weeks, and later the operation on the palate is performed.

I have called attention in the paper to the point to which Dr. Keen has referred. The difficulty he has had I have had in my earlier operations, and I could not account for it until I made a close examination. I then found that just at the line of junction between the horizontal plates of the palate bone there was a slight notch, and that was sufficient to prevent union at this point. In these cases I cut on each side and draw a segment of bone over, and in this way I have never failed to obtain union at this point.

I tried the operation of transplantation and closure by periosteal flaps in my earlier work. I have never succeeded satisfactorily with these methods, and therefore I have later used the method of Ferguson, making sections on each side and drawing the segments over the middle line. Where the alveolar process is very narrow it is difficult to get sufficient bone to close the gap, and if there is necrosis from splintering the reparative process is less likely to go on satisfactorily, but even when necrosis takes place reproduction of bone may occur.

Many surgeons look upon these operations as the *bête noire* of surgery. They do not like to undertake them. I am glad to say there has been a revival of the operation, particularly in Boston. I believe that the adaptation of any obturator is unsatisfactory. I have seen the obturator to which Dr. Keen refers, and I have found that patients complain of the discomfort arising from its use where it is applied to the soft palate. Closure of the hard palate by obturator is easily effected. I have not seen either abroad or in this country any obturator which gave the patient complete comfort, or which I thought as satisfactory as operation which closes the gap.

With regard to the results, we all know what they are. I have had some exceptional cases in which the results have been perfect, and I have had a large number in which there has been some improvement, and some in which there has been little or no improvement as regards articulation. I tell patients that a natural tone of voice cannot always be accomplished. This depends a good deal upon the size and form of the pharyngeal and post-nasal space. In some cases the pharynx is very capacious, and when an attempt is made to bring the soft palate in contact with the posterior wall of the pharynx the space is so great that this cannot be accomplished. In these cases the difficulty in articulation is not entirely removed, and sometimes the difficulty in deglutition is imperfect. In children I have found the greatest benefit derived from the moral effect produced by operation. I think the operation should be done and an effort made to help children suffering from the deformity.

DR. KEEN: I wish to say in regard to the obturator of Dr. Thorington, that although I have not seen it used in many cases, yet where it was used no discomfort was complained of. It has been so excellent that in cases of very large cleft I have rather determined to rely upon it instead of operation.

